# Supplementary material for: Decreased Expression of KLF4 Leading to Functional Deficit in Pediatric Patients with Intestinal Failure and Potential Therapeutic Strategy Using Decanoic Acid
Source: Nutrients. 2023 Jun 7;15(12):2660. doi: 10.3390/nu15122660 (PMC10305161; doi:10.3390/nu15122660)
Supplement: Supplementary file 1 [file nutrients-15-02660-s001.zip › nutrients-2371166-supplementary.pdf]

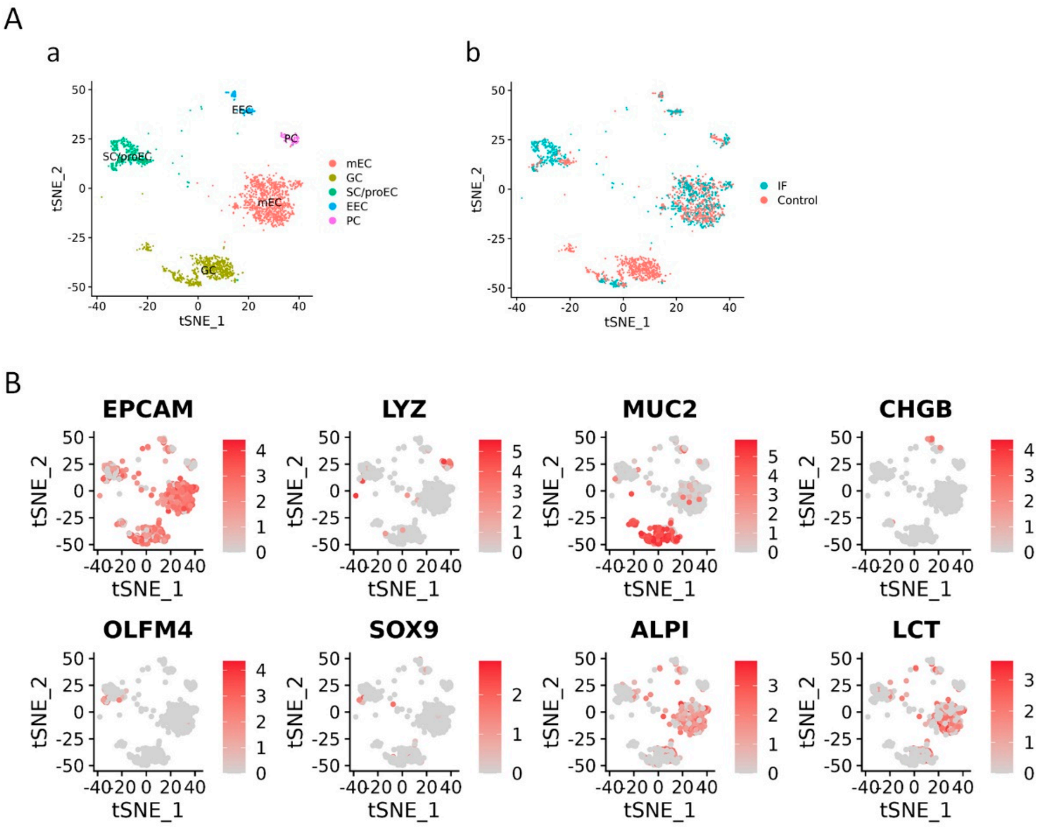

Figure S1. Enterocyte landscapes of IF patients. (**A**) t-SNE plots of single-cell clusters. In total, 4668 epithelial cells from 3 Control donors and 3717 epithelial cells from 3 IF donors were plotted by either cell types (**a**) or groups (**b**). (**B**) Projection of cell markers. EPCAM identifies all the epithelial cells, while LYZ, MUC2 and CHGB is one of the makers for Paneth cells (PC), Goblet cells (GC) and Enteroendocrine cells (EEC), respectively. OLFM4 and SOX9 were used to identify stem cells and enterocyte progenitors (SC/proEC). ALPI and LCT were used to identify mature enterocytes (mEC).

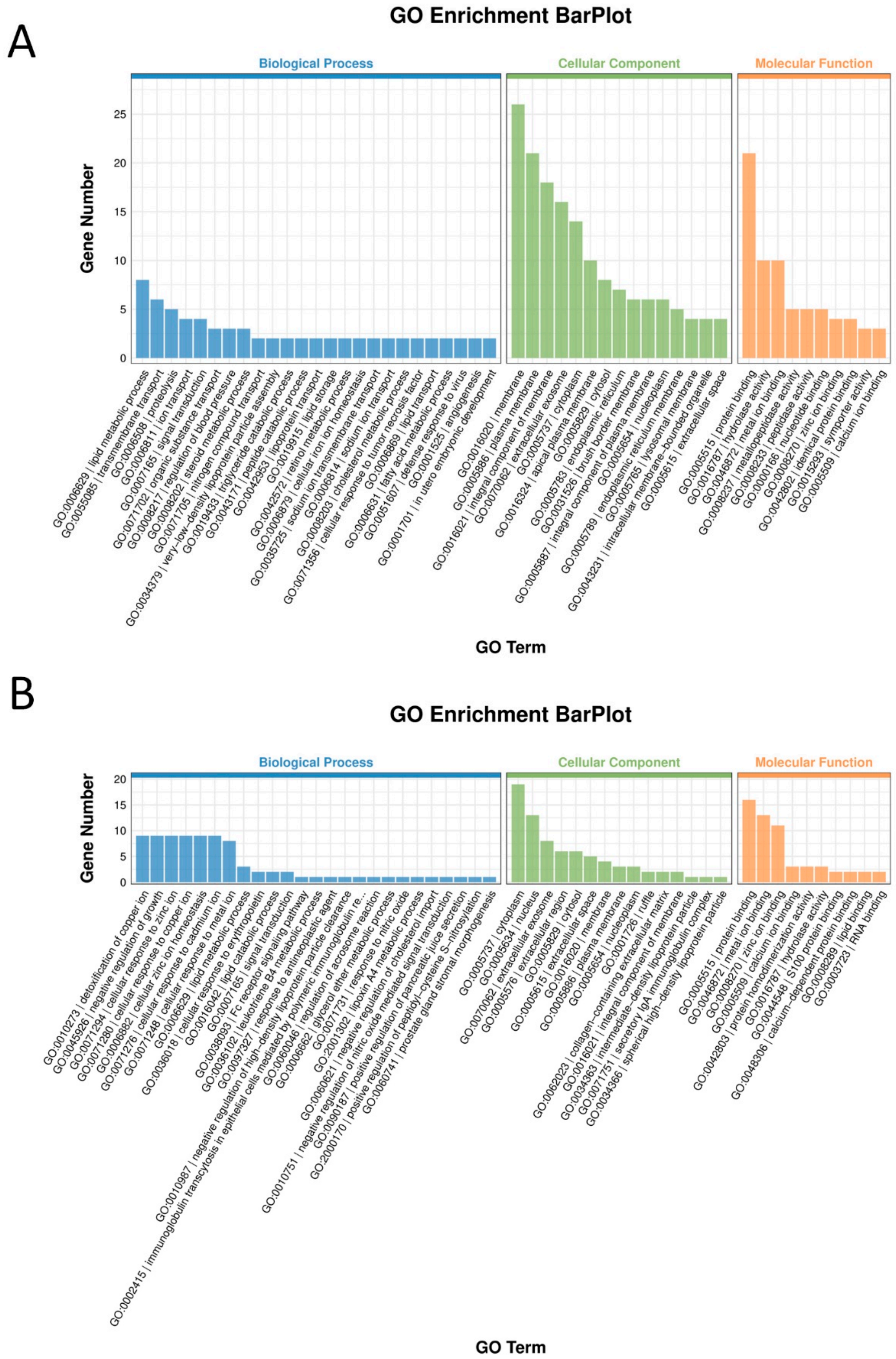

Figure S2. GO enrichment analysis showing main differences in the mature enterocytes. (**A**) GO enrichment of the genes down-regulated in IF patients. (**B**) GO enrichment of the genes up-regulated in IF patients.

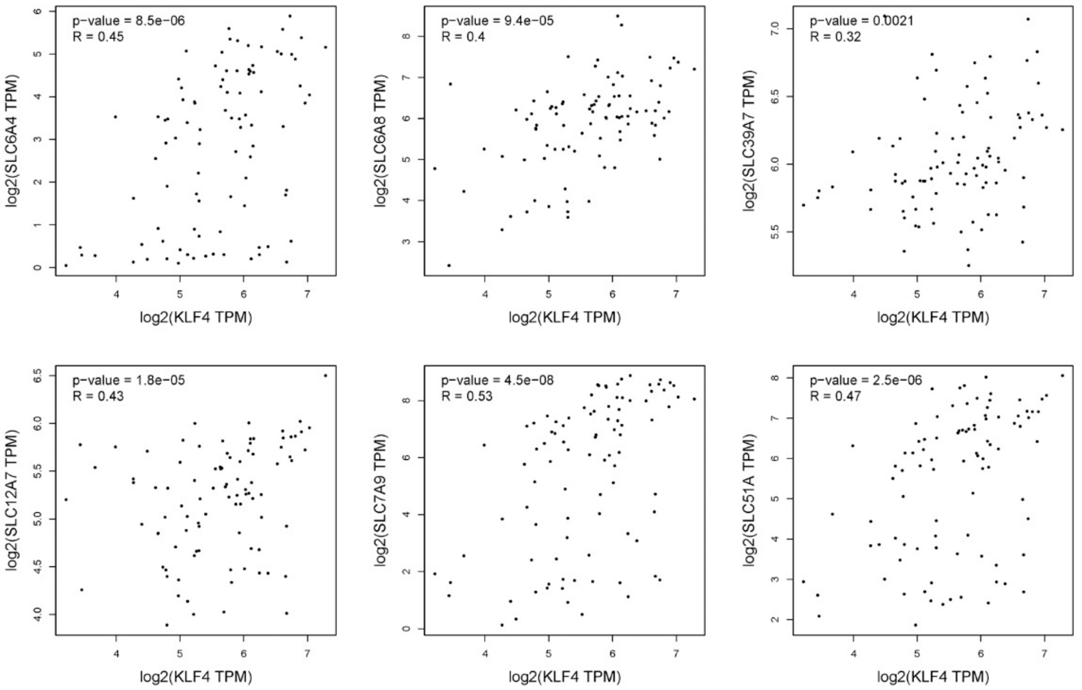

Figure S3. Correlation of KLF4 and SLC transporters. Data were extracted from the public database (GTEx) using normal terminal ileal tissues.

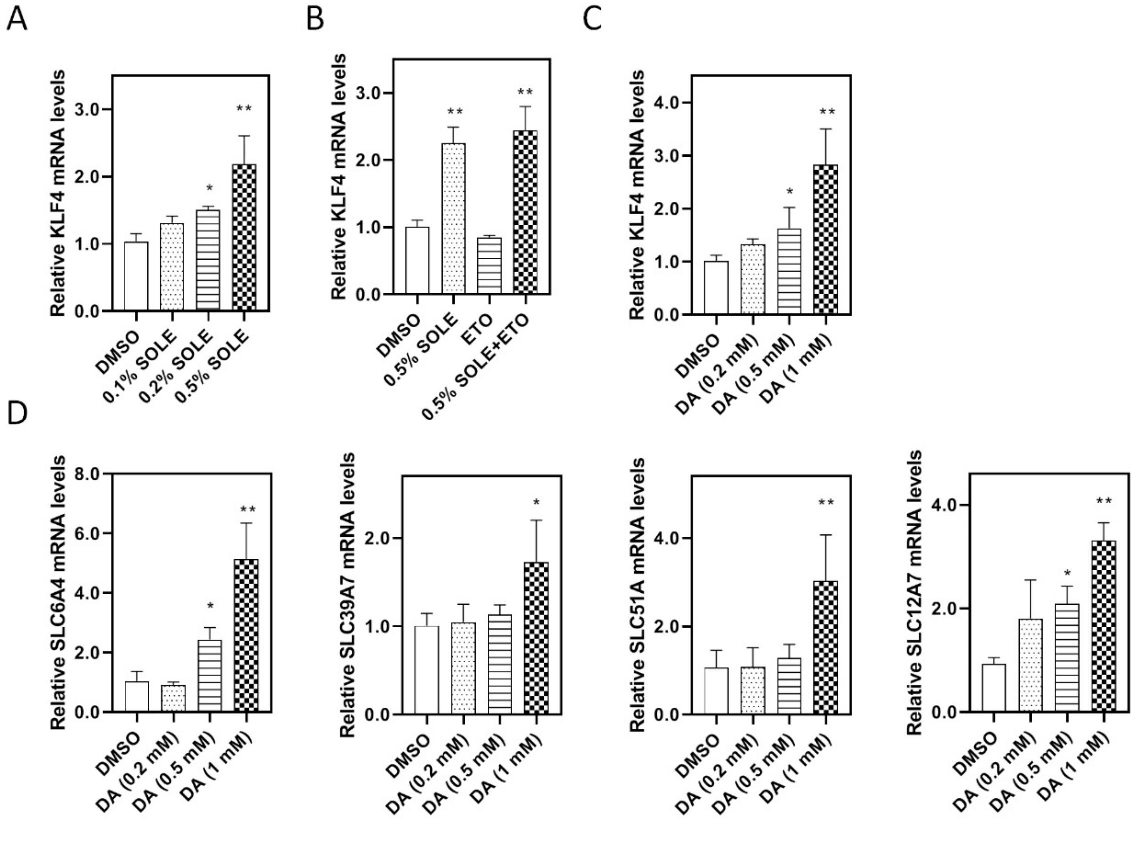

Figure S4. Effect of DA on the expression of KLF4 and SLC transporters. (**A**) KLF4 mRNA levels in Caco-2 cells treated with SOLE (**B**) KLF4 mRNA levels in Caco-2 cells treated with SOLE and ETO (**C**) KLF4 mRNA levels in Caco-2 cells treated with DA. (**D**) SLC transporter mRNA levels in Caco-2 cells treated with DA. Data are presented as mean ± SD. * $p < 0.05$, ** $p < 0.01$.
